# USING PHOTOVOICE TO EXPLORE THE STRUCTURED ENVIRONMENT AND ADVANCE POLICY IN MULTIPLE CONTEXTS

**DOI:** 10.1093/geroni/igac059.1491

**Published:** 2022-12-20

**Authors:** Melissa Hladek, Hae-Ra Han

## Abstract

Contextual factors, including social determinants of health, have gained recognition for greatly influencing aging trajectories and chronic disease progression. Although traditional qualitative research gives a rich understanding of the internal psychological experience, it is unable to assess socio-cultural and built environment aspects of life apart from the verbal descriptions from participants. This symposium examines the use of Photovoice as a tool to assess the socio-cultural and structural context surrounding the participant. This symposium will present a narrative review on the use of current Photovoice methodologies in dementia care; results from three studies using photovoice, one with participants with mild cognitive impairment and their caregivers to explore their perceptions of aging in their home, the second exploring the lived experience and structural barriers among older adults with pre-frailty or frailty awaiting kidney transplant, and the third exploring the older adult peer mentors’ experience providing community-delivered hearing care. The talks will also address practical aspects of using photovoice such as institutional review board tips, logistical challenges, interview preparation and coding approaches. The fifth talk will describe Photovoice result dissemination strategies, including photo exhibits and stakeholder meetings, used to engage the community and advance policy to improve disparities. Photovoice as a research tool originally aimed to reinforce empowerment for those marginalized in society to describe their lived environment. This important aim is augmented in this symposium by improving researchers’ understanding of context to design more impactful research.

